# Chikungunya infection: six years after, rheumatic morbidity and impaired quality of life persist!

**DOI:** 10.1186/1471-2334-14-S2-O21

**Published:** 2014-05-23

**Authors:** F Simon, J Ferraro, E Javelle, C Marimoutou

**Affiliations:** 1 Laveran Military Teaching Hospital, French Military Health Service, Marseille, France; 2 Army Center for Epidemiology and Public Health, Marseille, France

## Background and objective

In 2006, all the military policemen deployed in Reunion Island during the chikungunya outbreak were enquired, 25% self-declared chikungunya infection (CHIK+) and 19% had positive serology. In 2008, a self-questionnaire was sent to the same persons, 403 responded, 101 were CHIK+. The latter presented higher frequency of rheumatic disorders and significantly lower quality of life (QoL) than non-infected (CHIK-) responders, 30 months after the outbreak. The purpose of this study is to understand if the difference persisted at six years.

## Method

The 646 participants to the 2006 enquiry were sent self-questionnaires by postmail with informed consent to the present study and proposal of new biological testing. Only 609 could be reached, and 252 fulfilled the questionnaire: 81 CHIK+ (32%) and 171 CHIK-. The results are based on their declaration. QoL was estimated with the SF-36 scale.

## Results

CHIK+ patients declared higher health care consumption between 2008 and 2012 (more frequent general practitioners consultation and use of paracetamol). They complained of more frequent and intense joint pain (40% versus 22% at least once a week, 64% versus 38% moderate to intense pain), stiffness and swelling during the same period. They also report more frequent fatigue, headache and depress mood (respectively 60% versus 32%, 42% vs29% and 21vs 6%, p<0.001). Finally all dimension of SF36-QoL were significantly impaired in CHIK+ patients, reflecting social, physical and mental impact of the disease.

## Conclusion

Despite a possible selection bias of the most symptomatic CHIK+ patients increasing the observed differences, the large differences observed in rheumatic morbidity, fatigue, and QoL lead to the conclusion that CHIK infection has very long term impact on health, social life and QoL with a very low proportion of patients returning to their previous health status.

